# Ketorolac Administration After Colorectal Surgery Increases Anastomotic Leak Rate: A Meta-Analysis and Systematic Review

**DOI:** 10.3389/fsurg.2022.652806

**Published:** 2022-02-09

**Authors:** Wen Chen, Jing Liu, Yongqiang Yang, Yanhong Ai, Yueting Yang

**Affiliations:** ^1^Department of Anus and Intestine Surgery, Shijiazhuang People Hospital, Shijiazhuang, China; ^2^Department of Endocrinology, Hebei General Hospital, Shijiazhuang, China; ^3^Department of General Surgery, Shijiazhuang People Hospital, Shijiazhuang, China

**Keywords:** ketorolac, anastomotic leak, colorectal surgery, randomized controlled trials, meta-analysis

## Abstract

**Objective:**

This meta-analysis aimed to evaluate whether ketorolac administration is associated with an increased anastomotic leak rate after colorectal surgery.

**Methods:**

The literature was searched using the Web of Science, Embase, and PubMed databases, and the search ended on May 31, 2020. The Newcastle–Ottawa Scale was used to assess methodological quality. Statistical heterogeneity was assessed using the Chi-square *Q* test and *I*^2^ statistics. Subgroup analysis was performed, and Egger's test was used to assess publication bias.

**Results:**

This meta-analysis included seven studies with 400,822 patients. Our results demonstrated that ketorolac administration after surgery increases the risk of anastomotic leak [OR = 1.41, 95% CI: 0.81–2.49, *Z* = 1.21, *P* = 0.23]. Low heterogeneity was observed across these studies (*I*^2^ = 0%, *P* = 0.51). The results of subgroup analysis showed that the use of ketorolac in case–control and retrospective cohort studies significantly increased the risk of anastomotic leak (*P* < 0.05). Furthermore, the subgroup analysis revealed that ketorolac use increased anastomotic leak rate in patients in the United States and Canada, and ketorolac plus morphine use did not increase anastomotic leak rate in Taiwanese patients (*P* < 0.05). No significant publication bias was observed (*P* = 0.126). Moreover, the analysis of risk factors related to anastomotic leak rate indicated that the total use of ketorolac did not increase the risk of anastomotic leak similar to the control group (*P* > 0.05).

**Conclusion:**

The meta-analysis indicates that the use of ketorolac increases the risk of anastomotic leak after colorectal surgery.

**Systematic Review Registration:**

PROSPERO, identifier CRD42020195724.

## Introduction

Colorectal cancer affects more than 1.9 million people worldwide per year (1). Surgery is the most common treatment for colorectal cancer. Anastomotic leak after colorectal surgery is a serious postoperative complication that may be life-threatening. Communication between the hollow organ lumen and the peritoneal cavity at the level of the anastomosis is referred to as anastomotic leak (2). According to reports, the incidence of anastomotic leak in colorectal surgery varies from 1 to 19% (3, 4). Moreover, postoperative deaths related to complications of anastomotic leak account for approximately one-third of all deaths after colorectal cancer surgery (5). Currently, the physiological mechanism that underlies anastomotic fistula is unknown. Nonsteroidal anti-inflammatory drugs (NSAIDs) are analgesics that play an important role in opioid-sparing protocols (6, 7). The use of NSAIDs has been shown to reduce the length of hospital stay and the time to recover bowel function (8, 9). Ketorolac is a non-selective NSAID that can affect the formation of cyclooxygenase (COX) and thus reduce the production of prostaglandins. In many studies, its analgesic effect is stronger than that of other NSAIDs, such as tramadol and diclofenac (10–13). Therefore, ketorolac has been widely used in various types of postoperative analgesia for colorectal surgery (14–16).

However, some evidence suggested that a higher incidence of surgical complications (anastomotic leak) is associated with an increase in the use of ketorolac (17, 18). Recently, other studies have shown that ketorolac exposure is not associated with anastomotic leak during elective colorectal surgery (19, 20). Therefore, the purpose of this study was to determine whether the administration of ketorolac after colorectal surgery will increase the anastomotic leak rate, to provide a basis for clinicians to use ketorolac after colorectal surgery.

## Materials and Methods

### Data Source and Search Strategy

This meta-analysis was performed according to the Preferred Reporting Items for Systematic Reviews and Meta-Analyses (PRISMA) guidelines (Supplementary Table S1) and it was registered in PROSPERO with the registration number CRD42020195724.

The Web of Science, Embase, and PubMed databases were searched to identify related articles. There was no language restriction during the search, and the deadline was May 31, 2020. The literature was searched using a combination of free-text terms and MeSH terms; the main search terms were: “Ketorolac,” “Acular,” “Toradol,” “Colorectal surgery,” “Surgery Specialty,” and “Anastomotic leaks.” In addition, the reference list of related reviews was manually searched to identify more related articles.

### Eligibility Criteria

The following studies were included in the analysis: (1) studies that involved patients who underwent colonic or rectal resection with anastomosis; (2) studies that involved intervention and comparison as follows: (a) patients who received ketorolac and morphine must be compared to a control group that received morphine and (b) patients who received ketorolac must be compared to a control group that did not receive ketorolac; and (3) studies that provided the anastomotic leak rate data. Patients were regarded to have an anastomotic leak if it was documented during reoperation and/or it was clinically suspected and radiologically verified based on contrast leakage or abscess at the site of the anastomosis with or without percutaneous drainage. Besides, due to the limited number of studies currently available, both randomized controlled trials (RCTs) and non-randomized observational studies were included.

Animal-based research and research that did not involve gastrointestinal, colon, or rectal surgery; meeting abstracts, editorials, and case reports; and studies that did not have anastomotic leak rate as the outcome or studies involving interventions with drugs other than ketorolac in the treatment group were excluded.

### Data Extraction and Quality Assessment

Two investigators (Jing Liu and Wen Chen) independently searched, selected, and extracted publications from the databases used. Inconsistent data were discussed by the two investigators to reach a consensus. The Newcastle–Ottawa Scale (NOS) is a representative tool used to measure the quality of case–control or cohort studies. NOS includes three classifications: low quality (0–3), medium quality (4–6), and high quality (7–9, 21). The risk of bias in the included RCT studies was assessed independently by both reviewers using the Cochrane Collaboration's tool for assessing the risk of bias.

In addition, the following information was collected: first author's name, publication date, country in which the study was conducted, study design, number of cases and controls, intervention and control groups, average age, diagnosis, type of surgery, adjustment confounders, and adjusted odds ratios (OR) (95% confidence interval [CI]). All entries were confirmed by two authors (Jing Liu and Wen Chen) and examined at least two times to ensure accuracy and completeness.

### Statistical Analysis

Data were statistically analyzed using RevMan version 5.3. The multivariate-adjusted ORs and corresponding 95% CIs reported in the studies were used to produce forest plots, and the total dose of ketorolac was quantified using the weighted mean difference (WMD) with a 95% CI. Heterogeneity among different studies was quantified using *I*^2^. When *I*^2^ > 50%, which was considered to be highly heterogeneous, the randomized control model was used for analysis, and when *I*^2^ ≤ 50%, it was considered to have low heterogeneity, and the fixed model was used for analysis. The reasons for heterogeneity were explored using subgroup analyses. Publication bias was quantified using funnel plots and Egger's test. Differences were considered statistically significant at *P* < 0.05, which indicates that there is no publication bias.

## Results

### Search Results

**Figure 1:**
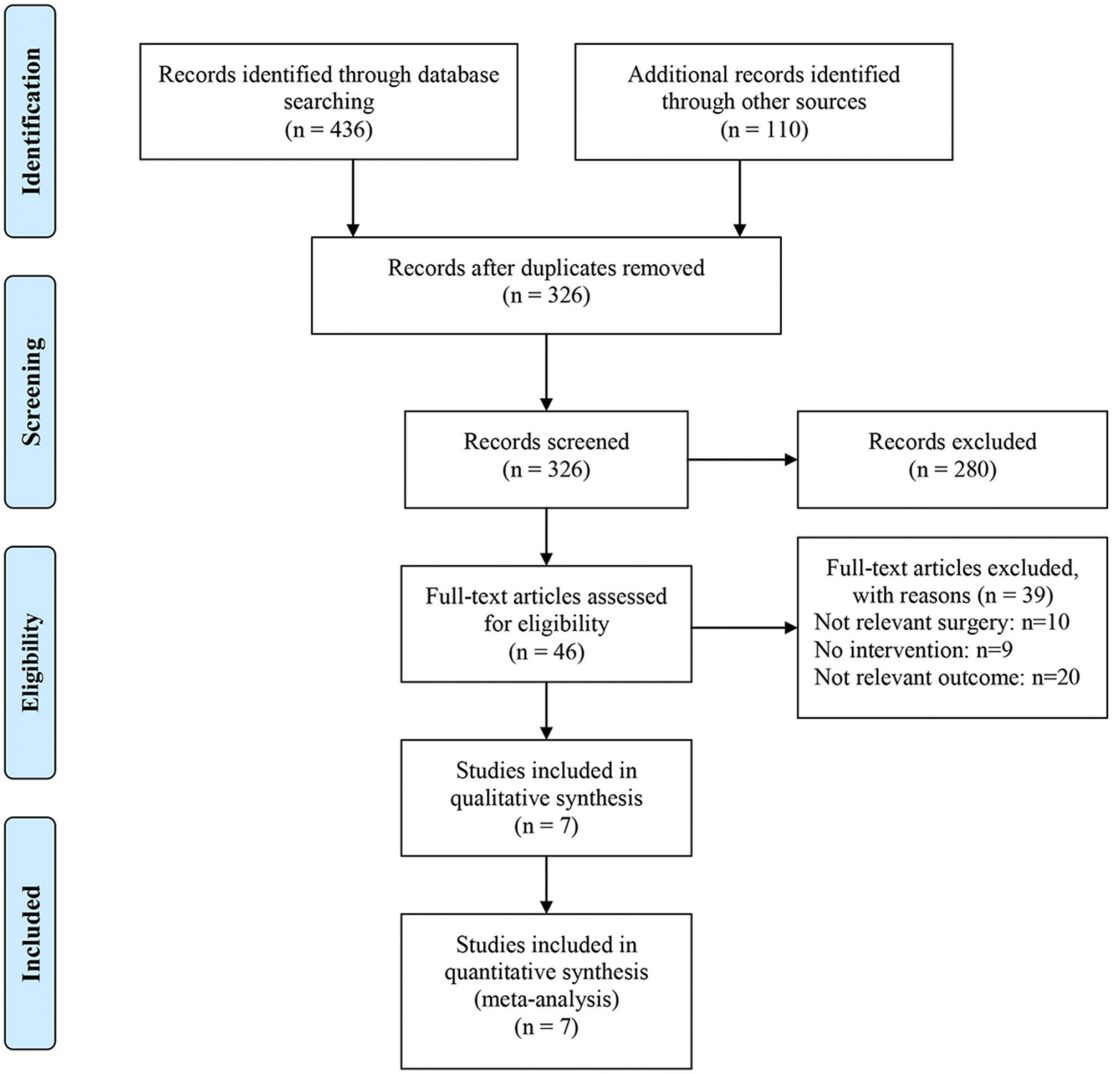
Flow chart of studies identified and included in the current meta-analysis.

A systematic search identified 546 eligible studies. After removing duplicate documents, 326 studies remained. Based on the title and abstract, 280 studies that did not meet the inclusion criteria were excluded. The remaining 46 studies were screened based on full-text reading. Ten of the 46 studies did not describe related surgery, nine did not use ketorolac for intervention, and 20 did not describe appropriate outcome measures. Finally, seven studies that met these requirements were included. A flow chart of the article selection process is shown in Figure 1. A total of 400,822 patients with colorectal cancer were included, including 20,929 and 379,893 patients in the intervention and control groups, respectively (Table 1). Two studies described patients who received either ketorolac plus intravenous patient-controlled analgesia (PCA) morphine (K+M) or intravenous PCA morphine (M) after elective colorectal resection (8, 9). One study compared patients who received ketorolac with those who received other NSAIDs (23). The other four studies compared the group that received ketorolac with the no ketorolac group (17, 19, 22, 24). With respect to their study methodology, three of the selected studies were RCTs, three were retrospective cohort studies, and one was a nested, matched case–control study.

**Table 1:** Brief characteristics of included studies.

**Reference**	**Study type**	**Race**	**Intervention (*n*)**	**Male / female**	**Average age**	**Weight (kg)**	**Diagnosis**	**Type of surgery**	**The number of anastomotic leak**	**Adjustment by**	**Adjusted OR(95% CI)**
Chen (8)	RCT	Taiwan	K+M (41) vs. M (38)	K+M (22/13) vs. M (19/20)	64.5 (48.5–71.0) vs. 68 (47.8–74.0)	61.1 ± 10.9 vs. 61 ± 13.4	/	K+M (Rectal surgery 7 and colon surgery 32) vs. M (Rectal surgery 8 and colon surgery 27)	K+M (2/41) vs. M(1/38)	/	OR 1.9 (0.17–21.82)
Schlachta (22)	RCT	Canada	K (22) vs. no K (22)	K (12/10) vs. no K (8/14)	59.5 ± 8.2 vs. 61.4 ± 12.4	77.5 ± 13.0 vs. 79.4 ± 17.0	/	/	K (4/22) vs. no K (1/22)	/	OR 4.67 (0.48–45.62)
Chen (9)	RCT	Taiwan	K+M (53) vs. M (56)	K+M (23/29) vs. M (30/20)	57.3 ± 11.6 vs. 60.5 ± 12.2	58.7 ± 11.2 vs. 62.2 ± 12.4	/	K+M (Rectal surgery 11 and colon surgery 39) vs. M [(Rectal surgery 10 and colon surgery 42)]	K+M (3/53) vs. M (1/56)	/	OR 3.3 (0.33–32.76)
Subendran (23)	Case -Control	Canada	K (131) vs. no K (131)	K (72/59) vs. no K (73/58)	47.0 ± 17.9 vs. 46.5 ± 17.8	/	K (colorectal cancer 45 and no cancer 86) vs. no K(colorectal cancer 45 and no cancer 86)	K (Ieal surgery 61, Rectal surgery 25 and colon surgery 45) vs. no K (Ieal surgery 61, Rectal surgery 25 and colon surgery 45)	K (68/131) vs. no K (63/131)	age, sex, year of surgery, type of surgery, underlying disease, use of preoperative steroids, smoking status, other comorbidities, total ketorolac dose, and method of detection of the anastomotic leak	OR 2.09 (1.12–3.89)
Saleh (24)	cohort	Canada	K (355) vs. no K (376)	K (186/169) vs. no K (230/146)	59.7 ± 13.5 vs. 66.9 ± 13.7	/	K (colorectal cancer 223 and no cancer 132) vs. no K (colorectal cancer 256 and no cancer 121)	K (Rectal surgery 126 and colon surgery 229) vs. no K (Rectal surgery 112 and colon surgery 264)	K (12/355) vs. no K (12/376)	Age, smoking,Steroid use	OR 1.21 (0.52–2.84)
Kotagal (17)	cohort	USA	K (19,780) vs. no K (37,8972)	K (6489/13291) vs. no K (171510/207462)	48 (39,56) vs. 52 (41,59)	/	K (colorectal cancer 718 and no cancer 19,062) vs. no K (colorectal cancer 21,812 and no cancer 357,160)	K(Rectal and colon surgery 11,622 and Noncolorectal GI Tract 8,158) vs. no K(Rectal and colon surgery 310,959 and Noncolorectal GI Tract 168,013)	/	demographic characteristics, comorbidities, surgery type/indication, and preoperative medications	OR 1.2 (1.06–1.36)
Hawkins (19)	cohort	USA	K (547) vs. no K (298)	K (256/291) vs. no K (127/171)	52.3 (37.6–61.)vs. 68.8 (60.1–75.3)	/	K (colorectal cancer 255 and no cancer 292) vs. no K (colorectal cancer 181 and no cancer 117)	K(Ieal surgery 278, Rectal surgery 116 and colon surgery 153) vs. no K(Ieal surgery 119, Rectal surgery 83 and colon surgery 96)	K (17/547) vs. no K (10/298)	sex, race/ethnicity, age, obesity (BMI≥30), and reason for procedure (neoplasia, IBD, and benign disease). Comorbidities included chronic corticosteroid or other immunosuppressant use within 30 days, diabetes mellitus with medical treatment, being a current smoker within 1 year, dyspnea, functional status, history of severe chronic obstructive pulmonary disease, weight loss >10%, hypertension requiring medication, and ASA physical status	OR 0.98 (0.38–2.57)

**Figure 2:**
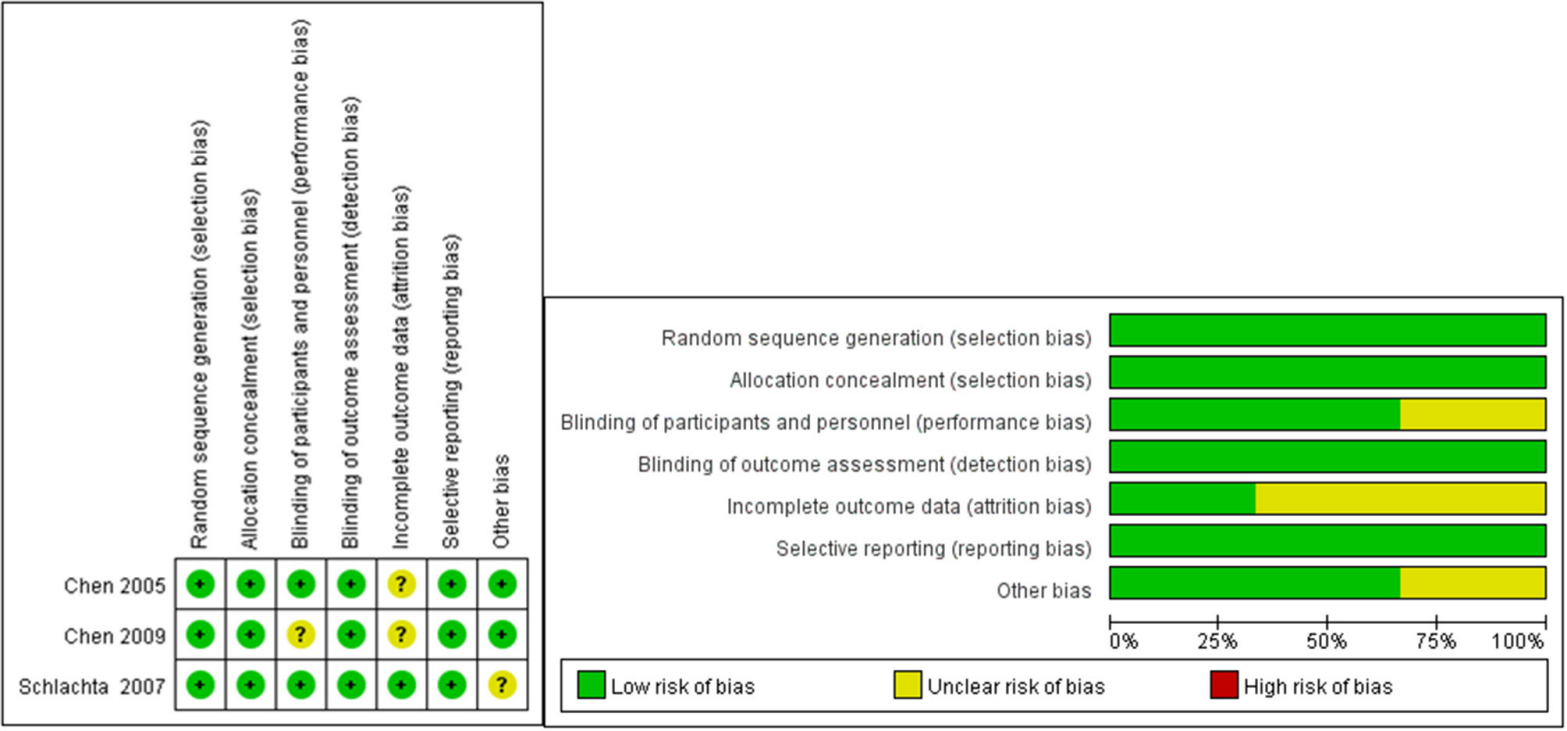
Risk of bias assessment of RCT studies.

The three cohort studies and one case report study were shown to be of moderate or high quality using the NOS (Tables 2, 3). Figure 2 shows that all RCT studies had a low risk of bias.

**Table 2:** Quality assessment of the case-control and cohort studies.

**First author**	**Representativeness of the cases**	**Case definition adequate**	**Ascertainment of exposure**	**Same method of ascertainment for cases and controls**	**Control for important factor or additional factor**	**Selection of Controls**	**Definition of Controls**	**Non-Response rate**	**Total quality scores**
Malecki	✰	–	✰	–	✰	✰	✰	✰	6

**Table 3:** Quality assessment of the cohort studies.

**First author**	**Year**	**Representativeness of the exposed cohort**	**Selection of the non exposed cohort**	**Ascertainment of exposure**	**Demonstration that outcome of interest was not present at start of study**	**Comparability of cohorts on the basis of the design or analysis**	**Assessment of outcome**	**Was follow-up long enough for outcomes to occur**	**Adequacy of follow up of cohorts**	**Total quality scores**
Saleh	2014	✰	✰	✰	✰	✰	✰	✰	✰	8
Kotagal	2016	✰	✰	✰	✰	✰	✰	✰	✰	8
Hawkins	2018	✰	–	✰	✰	✰	✰	✰	✰	7

### Ketorolac Use and Anastomotic Leak Rate

**Figure 3:**
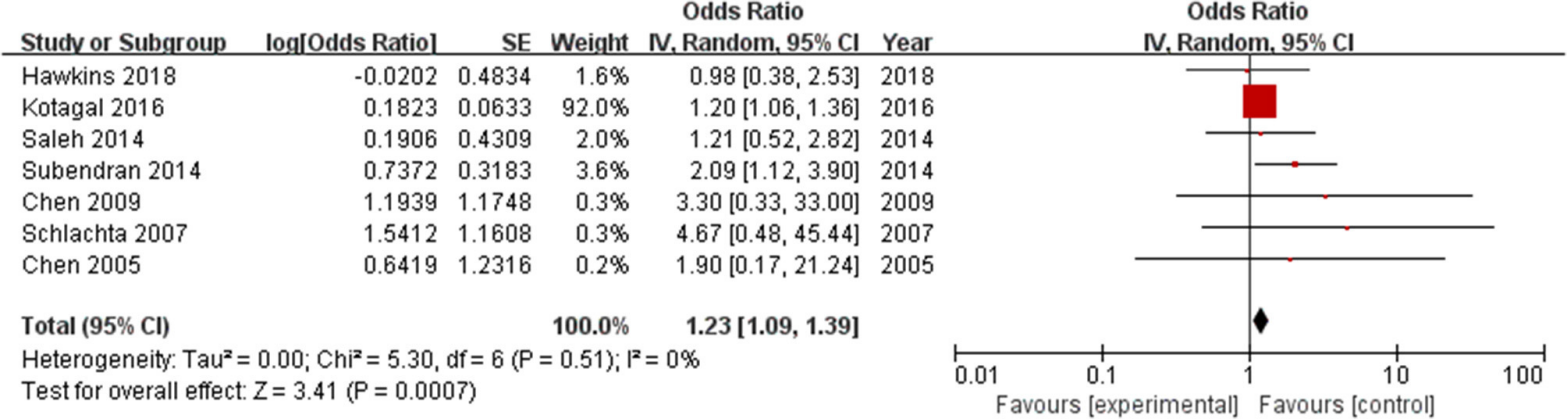
Forest plots of anastomotic leak rate in the intervention and control groups.

All included studies, which involved 400,822 patients, reported an anastomotic leak rate. The results showed that the use of ketorolac increased the risk of anastomotic leak in patients compared with the risk observed in the control group, and the difference was statistically significant (OR = 1.23, 95% CI = 1.09–1.39, *Z* = 3.41, *P* = 0.0007) (Figure 3). Heterogeneity, as defined by *I*^2^ statistics, was low (*I*^2^ = 0%, *P* = 0.51). Since this meta-analysis included fewer than 10 studies, no funnel plot was generated to assess publication bias. Egger's test showed no significant publication bias (*P* = 0.126).

**Figure 4:**
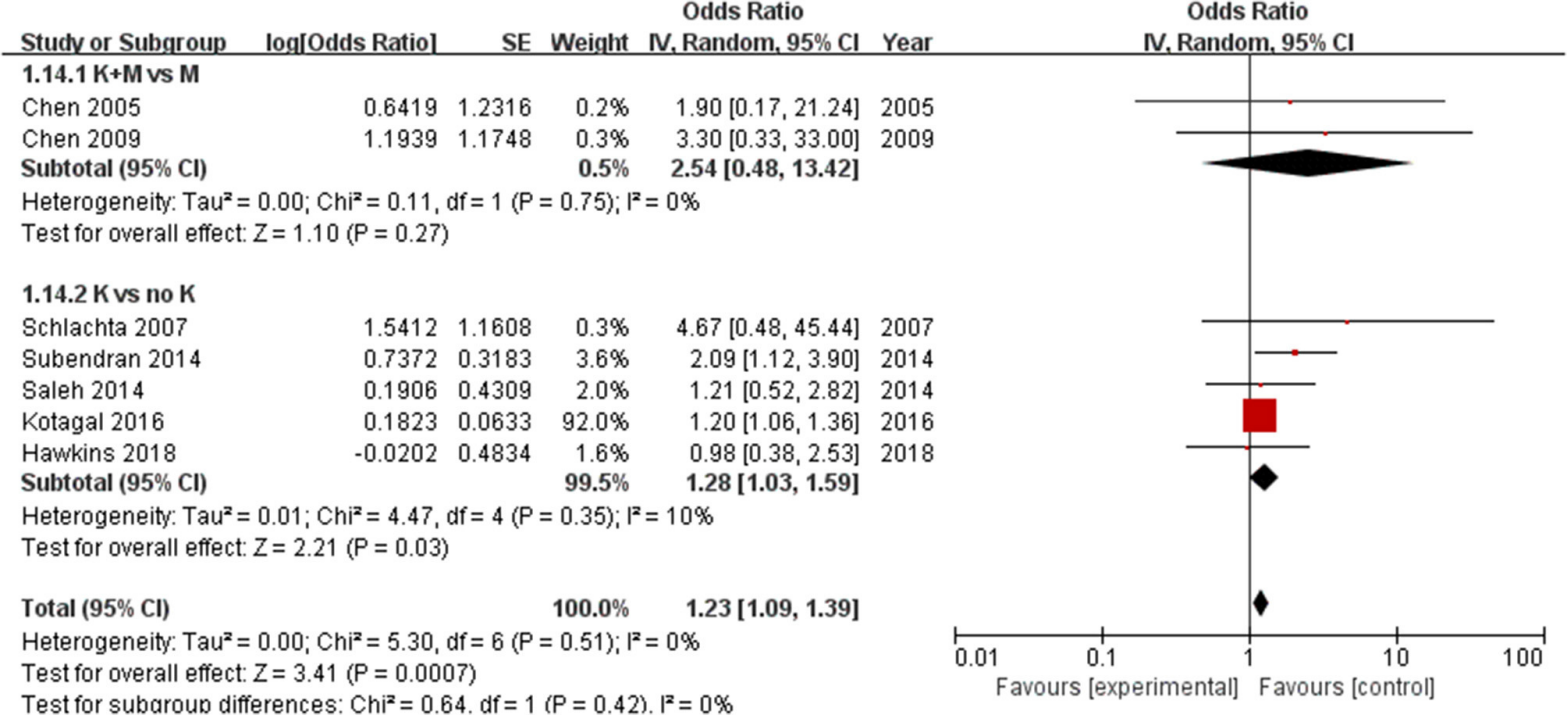
Forest plots of subgroups according to different study designs: 1.14.1 K+M (ketorolac plus morphine) *vs*. K (ketorolac); 1.14.2 K (ketorolac) *vs*. (no ketorolac).

The subgroup analysis based on different study designs showed that the risk of anastomotic leak was significantly increased with the use of ketorolac (*n* = 400,699) (OR = 1.28, 95% CI = 1.03–1.59, *P* = 0.03) with low heterogeneity (*I*^2^ = 10%). The addition of morphine to ketorolac (*n* = 123) can eliminate the risk of anastomotic leak (OR = 2.54, 95% CI = 0.48–13.42, *P* = 0.27) without significant heterogeneity (*I*^2^ = 0%) (Figure 4).

**Figure 5:**
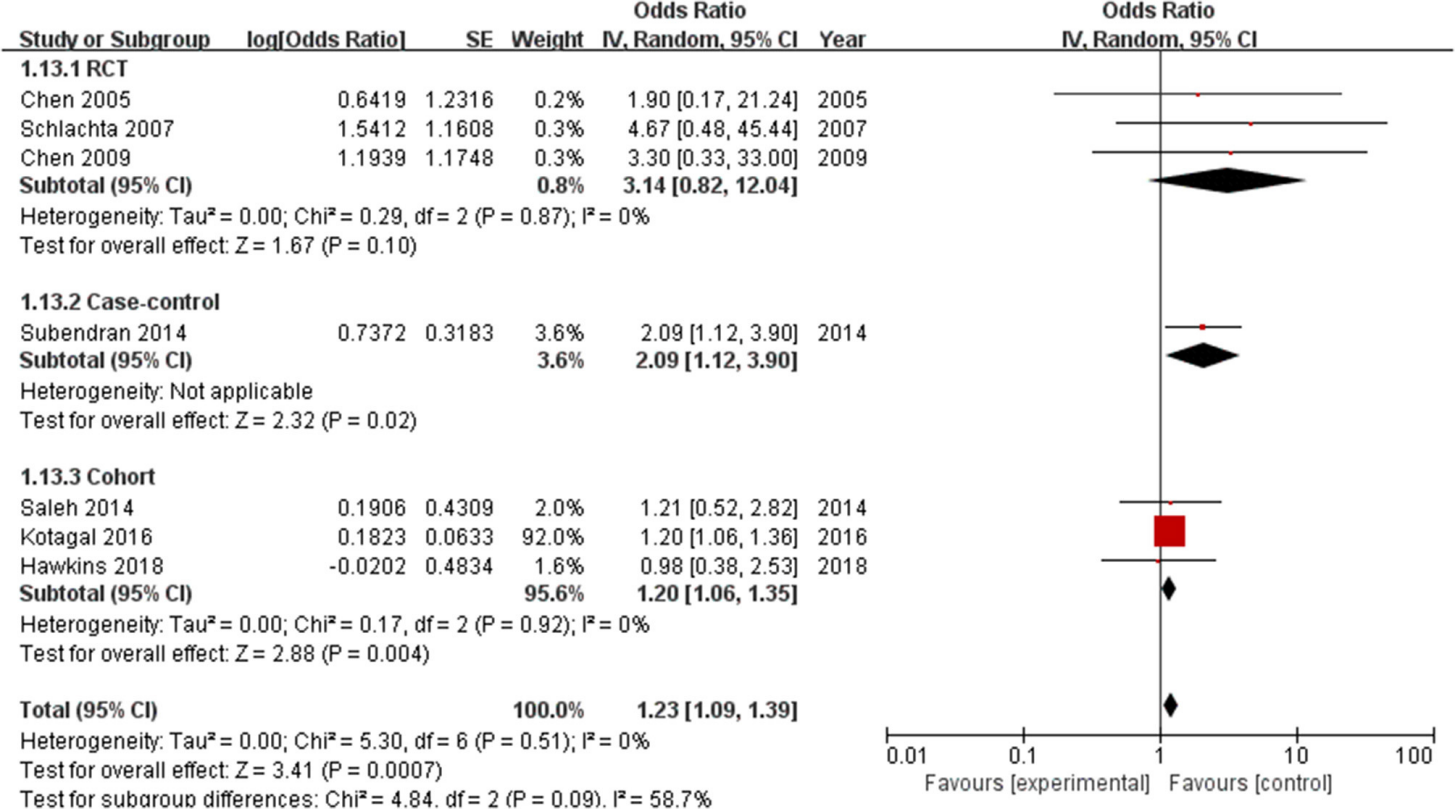
Forest plots of subgroups according to different experimental study designs.

Subgroup analysis was performed according to the type of study. The analysis of RCTs (including 232 patients) did not show a significant difference in the incidence of anastomotic leak between the ketorolac group and the control group (OR = 3.14, 95% CI = 0.82–12.04, *P* = 0.10). However, the risk of anastomotic leak was significantly increased in case–control (*n* = 262) (OR = 2.09, 95% CI = 1.12–3.9, *P* = 0.02) and retrospective cohort studies (*n* = 400,328) (OR = 1.2, 95% CI=1.06–1.35, *P* = 0.004) (Figure 5).

**Figure 6:**
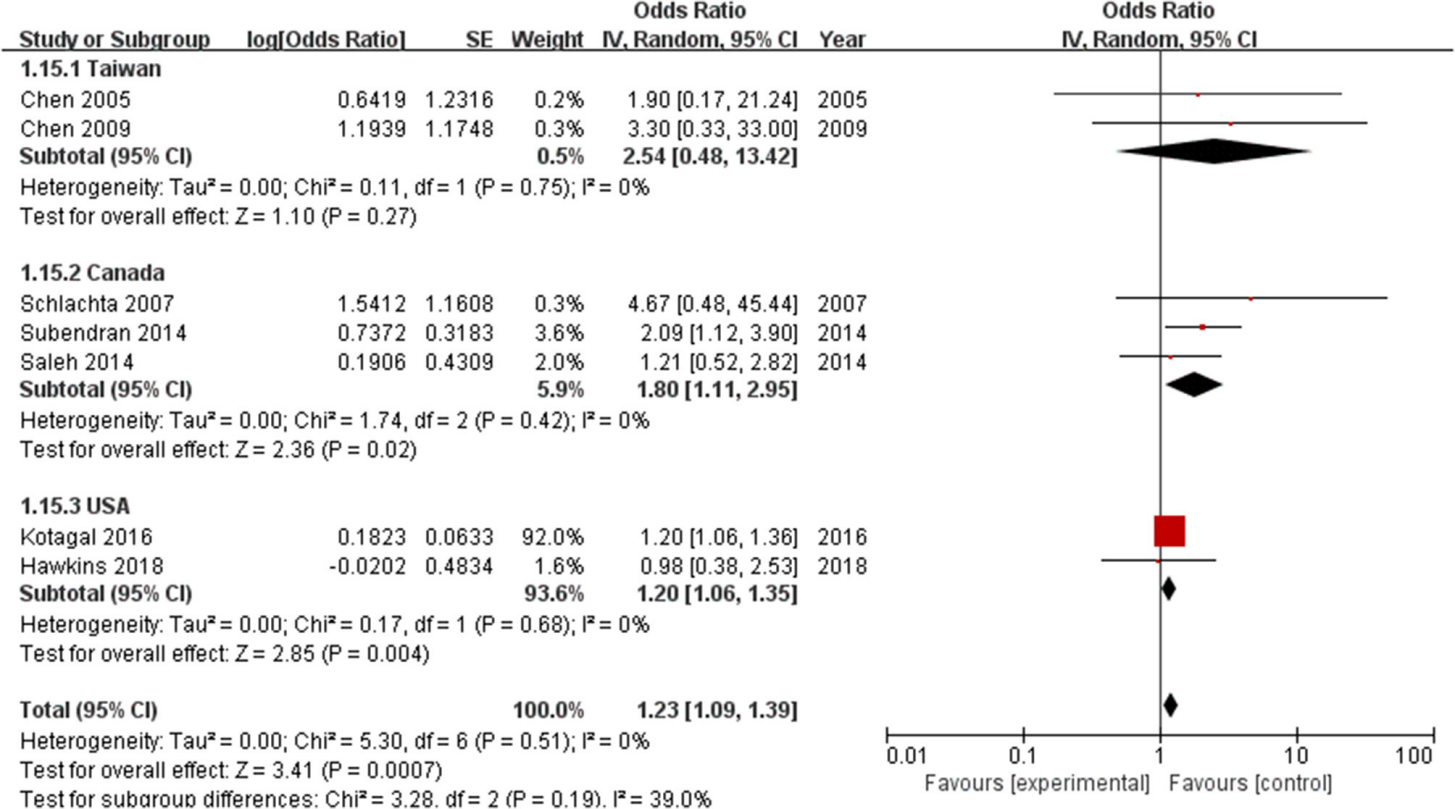
Forest plots of subgroups according to different geographical location.

Furthermore, a subgroup analysis of geographical location found that the incidence of anastomotic leak in Taiwanese patients (*n* = 188) was not significantly different between the ketorolac plus morphine group and the control group (OR = 2.54, 95% CI = 0.48–13.42, *Z* = 1.1, *P* = 0.27). However, this risk was observed in patients treated with ketorolac in Canada (*n* = 1,037) (OR = 1.80, 95% CI = 1.11–2.95, *Z* = 2.36, *P* = 0.02) and in the United States (*n* = 399,597) (OR = 1.20, 95% CI = 1.06–1.35, *Z* = 2.85, *P* = 0.004) (Figure 6).

### The Total Dose of Ketorolac Use and Anastomotic Leak Rate

**Figure 7:**
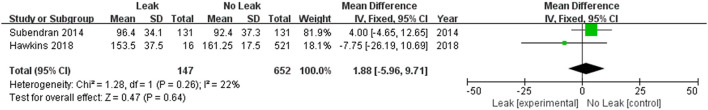
The relation between total dose of ketorolac use and anastomotic leak rate.

Only two studies (19, 23) reported the total dose of ketorolac use, and these studies involved 1,107 patients. No difference was found between the total dose of ketorolac and the incidence of anastomotic leak (OR = 1.88, 95% CI = −5.969.71, *Z* = 0.47, *P* = 0.64) (*I*^2^ = 22%, *P* = 0.26) (Figure 7).

### Sensitivity Analysis

Sensitivity analysis was used to evaluate the stability of the results. The significance of the results did not change after deleting one study at a time, indicating that the combined OR was relatively stable.

## Discussion

Overall, the evidence from this study suggests that ketorolac is associated with an increased risk of anastomotic leak after colorectal surgery. Besides, further subgroup analysis revealed that this effect varied between the use of ketorolac alone and the use of ketorolac plus morphine.

Nonsteroidal anti-inflammatory drugs are a powerful class of analgesics that are an important part of the multimodal approach used in ERAS programs to control postoperative pain (25). They operate as analgesics by inhibiting the activity of cyclooxygenase (COX) enzyme 2 (COX-2 selective) or both COX-1 and COX-2 enzyme activity (non-selective). Ketorolac is a non-specific COX inhibitor and an injectable non-steroidal anti-inflammatory drug with a good analgesic effect. This meta-analysis is the first to study the anastomotic leak rate associated with ketorolac use in patients undergoing colorectal surgery. A previous meta-analysis has examined the effect of NSAIDs on the healing of anastomoses after colorectal surgery (26, 27). Modasi et al. discovered that post-colorectal surgery NSAID administration increases anastomotic leak rate, while Arron et al. discovered that post-colorectal cancer surgery NSAID administration does not increase anastomotic leak rate (26, 28). There is still controversy over whether post-colorectal surgery NSAID administration will increase the anastomotic leak rate, and more clinical studies are needed to verify it. Arron et al. also found that neither non-selective NSAID use nor COX-2 selective NSAID use caused an increased anastomotic leak rate (28). Besides, Huang et al. and Modasi et al. discovered that ketorolac was not associated with an increase in leak rate; however, their meta-analyses only included 2–3 studies on ketorolac (26, 27). In contrast to their findings, this study discovered that ketorolac was correlated with an increase in anastomotic leak. Some studies have reported that the increased risk of anastomotic leak is related to certain risk factors (such as the male sex, obesity, drug dosage, and smoking) (29–32). Three of the included studies did not provide data after adjusting for risk factors, which may be the reason for the positive associations.

Furthermore, a subgroup analysis was performed on the study type, and an association between ketorolac and the anastomotic leak was observed in case–control and retrospective cohort studies, but not in RCT studies. In addition, the use of ketorolac was associated with an increased risk of anastomotic leak in patients in the United States and Canada, but not in Taiwanese patients. Some studies reported that there is a dose-dependent relationship between ketorolac administration and anastomotic leak (10, 33). This is inconsistent with the results of the present study. We did not find a relationship between the ketorolac dose and the leak. This may be because we only included two studies that reported the relationship between the dose of ketorolac and leak, and further research is needed to verify our findings.

Anastomotic leak is a serious complication that occurs after colorectal surgery, which can lead to increased morbidity and mortality (34–36). Non-selective NSAIDs (such as ketorolac) may affect the healing of the intestine by inhibiting the action of cyclooxygenase (37, 38). NSAIDs have been shown to weaken granulocyte function, which is an essential part of the acute phase of wound healing (39, 40). NSAIDs may also inhibit epithelial cell migration and mucosal recovery, which are important in the pathophysiology of intestinal ulcer healing (41). These findings suggest a potential biological mechanism that may explain the association identified in this study.

This meta-analysis has several limitations. First, the included studies were not RCTs; therefore, they may have been affected by potential bias. Second, the confounding factors adjusted in each study were different, and some unadjusted confounding factors may exist in some original studies. In addition, there may be other factors that caused anastomotic leaks in the included studies. Third, Fjederholt et al. (18) showed that the use of ketorolac in men results in a higher risk of an anastomotic leak than in women. Unfortunately, none of the included studies provided data after adjusting for all the risk factors. Fourth, two of the included studies have a large sample size. Although sensitivity analysis has been conducted and it is found that these two studies will not reverse the overall results, more high-quality studies are needed to verify these findings.

In conclusion, our findings indicated that ketorolac exposure is associated with the anastomotic leak, and the use of ketorolac increases the risk of anastomotic leak. Therefore, the use of ketorolac after colorectal surgery should be done with caution after weighing the potential risks and benefits.

## Data Availability Statement

The original contributions presented in the study are included in the article/Supplementary Material, further inquiries can be directed to the corresponding author.

## Author Contributions

WC and JL contributed to writing the manuscript. YqY and YhA performed the data search and analysis. YtY designed the study. All the authors corrected and improved the final text, read and approved the final manuscript.

## Funding

This work was supported by the Shijiazhuang Scientific and Technological Research and Development Guidance Plan (Grant Number: 191460883).

## Conflict of Interest

The authors declare that the research was conducted in the absence of any commercial or financial relationships that could be construed as a potential conflict of interest.

## Publisher's Note

All claims expressed in this article are solely those of the authors and do not necessarily represent those of their affiliated organizations, or those of the publisher, the editors and the reviewers. Any product that may be evaluated in this article, or claim that may be made by its manufacturer, is not guaranteed or endorsed by the publisher.

## References

[1] Sung H Ferlay J Siegel RL Laversanne M Soerjomataram I Jemal A . Global cancer statistics 2020: GLOBOCAN estimates of incidence and mortality worldwide for 36 cancers in 185 countries. CA Cancer J Clin. (2021) 71:209–49. 10.3322/caac.21660 33538338

[2] Rahbari NN Weitz J Hohenberger W Heald RJ Moran B Ulrich A . Definition and grading of anastomotic leakage following anterior resection of the rectum: a proposal by the International Study Group of Rectal Cancer. Surgery. (2010) 147:339–51. 10.1016/j.surg.2009.10.012 20004450

[3] Rickert A Willeke F Kienle P Post S. Management and outcome of anastomotic leakage after colonic surgery. Colorectal disease. (2010) 12:e216–223. 10.1111/j.1463-1318.2009.02152.x 20002697

[4] Vasiliu EC Zarnescu NO Costea R Neagu S. Review of Risk Factors for Anastomotic Leakage in Colorectal Surgery. Chirurgia. (2015) 110:319–26. 26305194

[5] Alberts JC Parvaiz A Moran BJ. Predicting risk and diminishing the consequences of anastomotic dehiscence following rectal resection. Colorectal disease. (2003) 5:478–82. 10.1046/j.1463-1318.2003.00515.x 12925084

[6] Maslin B Lipana L Roth B Kodumudi G Vadivelu N. Safety Considerations in the Use of Ketorolac for Postoperative Pain. Curr Drug Saf . (2017) 12:67–73. 10.2174/1574886311666160719154420 27440142

[7] Wick EC Grant MC Wu CL. Postoperative multimodal analgesia pain management with nonopioid analgesics and techniques: a review. JAMA Surg. (2017) 152:691–7. 10.1001/jamasurg.2017.0898 28564673

[8] Chen JY Wu GJ Mok MS Chou YH Sun WZ Chen PL . Effect of adding ketorolac to intravenous morphine patient-controlled analgesia on bowel function in colorectal surgery patients–a prospective, randomized, double-blind study. Acta Anaesthesiol Scand. (2005) 49:546–51. 10.1111/j.1399-6576.2005.00674.x 15777304

[9] Chen JY Ko TL Wen YR Wu SC Chou YH Yien HW . Opioid-sparing effects of ketorolac and its correlation with the recovery of postoperative bowel function in colorectal surgery patients: a prospective randomized double-blinded study. Clin J Pain. (2009) 25:485–9. 10.1097/AJP.0b013e31819a506b 19542795

[10] Volkow N Benveniste H McLellan AT. Use and Misuse of Opioids in Chronic Pain. Annu Rev Med. (2018) 69:451–65. 10.1146/annurev-med-011817-044739 29029586

[11] Gutta R Koehn CR James LE. Does ketorolac have a preemptive analgesic effect? A randomized, double-blind, control study. J Oral Maxillofac Surg. (2013) 71:2029–34. 10.1016/j.joms.2013.06.220 23993224

[12] Bugada D Lavand'homme P Ambrosoli AL Klersy C Braschi A Fanelli G . Effect of postoperative analgesia on acute and persistent postherniotomy pain: a randomized study. J Clin Anesth. (2015) 27:658–64. 10.1016/j.jclinane.2015.06.008 26329661

[13] Mohammadpour M Heidari Z Molani R. Comparison between diclofenac and ketorolac ophthalmic drops for pain management after photorefractive keratectomy: a randomized clinical study. Eye Contact Lens. (2019) 45:137–40. 10.1097/ICL.0000000000000524 29944510

[14] Manworren RC McElligott CD Deraska PV Santanelli J Blair S Ruscher KA . Efficacy of analgesic treatments to manage children's postoperative pain after laparoscopic appendectomy: retrospective medical record review. AORN journal. (2016) 103:317.e311. 10.1016/j.aorn.2016.01.013 26924376

[15] Murdoch J Ramsey G Day AG McMullen M Orr E Phelan R . Intraperitoneal ketorolac for post-cholecystectomy pain: a double-blind randomized-controlled trial. Can J Anaesth. (2016) 63:701–8. 10.1007/s12630-016-0611-4 26864193

[16] Hariri K Hechenbleikner E Dong M Kini SU Fernandez-Ranvier G Herron DM. Ketorolac use shortens hospital length of stay after bariatric surgery: a single-center 5-year experience. Obesity surgery. (2019) 29:2360–6. 10.1007/s11695-018-03636-z 31190264

[17] Kotagal M Hakkarainen TW Simianu VV Beck SJ Alfonso-Cristancho R Flum DR. Ketorolac use and postoperative complications in gastrointestinal surgery. Ann Surg. (2016) 263:71–5. 10.1097/SLA.0000000000001260 26106831 PMC4684464

[18] Fjederholt KT Okholm C Svendsen LB Achiam MP Kirkegård J Mortensen FV. Ketorolac and other NSAIDs increase the risk of anastomotic leakage after surgery for GEJ cancers: a cohort study of 557 patients. J Gastrointest Surg. (2018) 22:587–94. 10.1007/s11605-017-3623-7 29134504

[19] Hawkins AT McEvoy MD Wanderer JP Ford MM Hopkins MB Muldoon RL . Ketorolac use and anastomotic leak in elective colorectal surgery: a detailed analysis. Dis Colon Rectum. (2018) 61:1426–34. 10.1097/DCR.0000000000001244 30371548

[20] Corsini EM Hofstetter WL. Ketorolac use and anastomotic leak in patients with esophageal cancer. J Thorac Cardiovasc Surg. (2020) 161:448–54. 10.1016/j.jtcvs.2020.02.133 32340809

[21] A S. Critical evaluation of the Newcastle-Ottawa scale for the assessment of the quality of non-randomized studies in meta-analyses. Eur J Epidemiol. (2010) 25:603–5. 10.1007/s10654-010-9491-z 20652370

[22] Schlachta CM Burpee SE Fernandez C Chan B Mamazza J Poulin EC. Optimizing recovery after laparoscopic colon surgery (ORAL-CS): effect of intravenous ketorolac on length of hospital stay. Surg Endosc. (2007) 21:2212–9. 10.1007/s00464-007-9335-4 17440782

[23] Subendran J Siddiqui N Victor JC McLeod RS Govindarajan A NSAID. use and anastomotic leaks following elective colorectal surgery: a matched case-control study. J Gastrointest Surg. (2014) 18:1391–7. 10.1007/s11605-014-2563-8 24912916

[24] Saleh F Jackson TD Ambrosini L Gnanasegaram JJ Kwong J Quereshy F . Perioperative nonselective non-steroidal anti-inflammatory drugs are not associated with anastomotic leakage after colorectal surgery. J Gastrointest Surg. (2014) 18:1398–404. 10.1007/s11605-014-2486-4 24912914

[25] Gustafsson UO Scott MJ Hubner M Nygren J Demartines N Francis N . Guidelines for Perioperative care in elective colorectal surgery: enhanced recovery after surgery (ERAS((R))) society recommendations: 2018. World J Surg. (2019) 43:659–95. 10.1007/s00268-018-4844-y 30426190

[26] Modasi A Pace D Godwin M Smith C Curtis B. NSAID administration post colorectal surgery increases anastomotic leak rate: systematic review/meta-analysis. Surg Endosc. (2019) 33:879–85. 10.1007/s00464-018-6355-1 29998389

[27] Huang Y Tang SR Young CJ. Nonsteroidal anti-inflammatory drugs and anastomotic dehiscence after colorectal surgery: a meta-analysis. ANZ J Surg. (2018) 88:959–65. 10.1111/ans.14322 29164809

[28] Arron MNN Lier EJ de Wilt JHW Stommel MWJ van Goor H Ten Broek RPG. Postoperative administration of non-steroidal anti-inflammatory drugs in colorectal cancer surgery does not increase anastomotic leak rate; a systematic review and meta-analysis. Eur J Surg Oncol. (2020) 46:2167–73. 10.1016/j.ejso.2020.07.017 32792221

[29] Fischer C Lingsma H Hardwick R Cromwell DA Steyerberg E Groene O. Risk adjustment models for short-term outcomes after surgical resection for oesophagogastric cancer. Br J Surg. (2016) 103:105–16. 10.1002/bjs.9968 26607783

[30] Frasson M Flor-Lorente B Rodríguez JL Granero-Castro P Hervás D Alvarez Rico MA . Risk factors for anastomotic leak after colon resection for cancer: multivariate analysis and nomogram from a multicentric, prospective, national study with 3193 patients. Ann Surg. (2015) 262:321–30. 10.1097/SLA.0000000000000973 25361221

[31] Van Daele E Van de Putte D Ceelen W Van Nieuwenhove Y Pattyn P. Risk factors and consequences of anastomotic leakage after Ivor Lewis oesophagectomy†. Interact Cardiovasc Thorac Surg. (2016) 22:32–7. 10.1093/icvts/ivv276 26433973

[32] Richards CH Campbell V Ho C Hayes J Elliott T Thompson-Fawcett M. Smoking is a major risk factor for anastomotic leak in patients undergoing low anterior resection. Colorectal disease. (2012) 14:628–33. 10.1111/j.1463-1318.2011.02718.x 21749605

[33] Gorissen KJ Benning D Berghmans T Snoeijs MG Sosef MN Hulsewe KW . Risk of anastomotic leakage with non-steroidal anti-inflammatory drugs in colorectal surgery. Br J Surg. (2012) 99:721–7. 10.1002/bjs.8691 22318712

[34] Fielding LP Stewart-Brown S Blesovsky L Kearney G. Anastomotic integrity after operations for large-bowel cancer: a multicentre study. Br Med J. (1980) 281:411–4. 10.1136/bmj.281.6237.411 7427298 PMC1713296

[35] Fatouros MS Vekinis G Bourantas KL Mylonakis EP Scopelitou AS Malamou-Mitsis VD . Influence of growth factors erythropoietin and granulocyte macrophage colony stimulating factor on mechanical strength and healing of colonic anastomoses in rats. Eur J Surg. (1999) 165:986–92. 10.1080/110241599750008143 10574109

[36] Kiyama T Onda M Tokunaga A Yoshiyuki T Barbul A. Effect of early postoperative feeding on the healing of colonic anastomoses in the presence of intra-abdominal sepsis in rats. Dis Colon Rectum. (2000) 43:S54–58. 10.1007/BF02237227 11052479

[37] Chen MR Dragoo JL. The effect of nonsteroidal anti-inflammatory drugs on tissue healing. Knee Surg Sports Traumatol Arthrosc. (2013) 21:540–9. 10.1007/s00167-012-2095-2 22744434

[38] Hakkarainen TW Steele SR Bastaworous A Dellinger EP Farrokhi E Farjah F . Nonsteroidal anti-inflammatory drugs and the risk for anastomotic failure: a report from Washington state's surgical care and outcomes assessment program (SCOAP). JAMA Surg. (2015) 150:223–8. 10.1001/jamasurg.2014.2239 25607250 PMC4524521

[39] Busti AJ Hooper JS Amaya CJ Kazi S. Effects of perioperative antiinflammatory and immunomodulating therapy on surgical wound healing. Pharmacotherapy. (2005) 25:1566–91. 10.1592/phco.2005.25.11.1566 16232020

[40] Stevens DL. Could nonsteroidal antiinflammatory drugs (NSAIDs) enhance the progression of bacterial infections to toxic shock syndrome? Clin Infect Dis. (1995) 21:977–80. 10.1093/clinids/21.4.977 8645850

[41] Freeman LC Narvaez DF McCoy A von Stein FB Young S Silver K . Depolarization and decreased surface expression of K+ channels contribute to NSAID-inhibition of intestinal restitution. Biochem Pharmacol. (2007) 74:74–85. 10.1016/j.bcp.2007.03.030 17499219 PMC3269908

